# Using UHF RFID Properties to Develop and Optimize an Upper-Limb Rehabilitation System

**DOI:** 10.3390/s20113224

**Published:** 2020-06-05

**Authors:** Walter Baccinelli, Maria Bulgheroni, Carlo Albino Frigo

**Affiliations:** 1 R&D Department, Ab.Acus Srl, via F. Caracciolo 77, 20155 Milano, Italy; mariabulgheroni@ab-acus.com; 2 Movement Biomechanics and Motor Control Lab, DEIB, Politecnico di Milano, 20133 Milano, Italy; carlo.frigo@polimi.it

**Keywords:** rehabilitation, RFID, upper limb, movement analysis

## Abstract

Rehabilitation of the upper limb is an important aspect of the therapy for people affected by neuromotor diseases for the recovery of the capability to perform activities of daily living (ADLs). Nonetheless, the costs associated with the administration of rehabilitation therapy and the increasing number of patients highlight the need for new solutions. Technology-based solutions and, in particular, telerehabilitation could strongly impact in this field. In this paper, a new system based on radiofrequency (RF) technology is presented which is able to effectively provide home-based telerehabilitation and extract meaningful information on the therapy execution performance. The technology has been tuned to the needs of the rehabilitation system, optimizing the hardware, the communication protocol and the software control. A methodology for extracting the execution time of the rehabilitation tasks, the distance covered by the patient’s hand in each subtask and the velocity profile is presented. The results show that a highly usable system for the rehabilitation of the upper limb has been developed using the RF technology and that performance metrics can be reliably extracted by the acquired signals.

## 1. Introduction

One of the most important goals of the rehabilitation process in neuromotor diseases is the recovery of upper limb functionalities. The presence of upper limb motor impairments has been shown to be related to anxiety, poor emotional reactions and social isolation [1], due to the impossibility to perform activities of daily living (ADLs). Indeed, such activities have an impact on the upper limbs’ functionalities [2], reducing arm and hand motor performances and thus highly impacting independence and quality of life. Providing effective rehabilitation strategies and tools for addressing upper limb impairments is thus essential for enhancing the quality of life. The importance of focusing on upper limbs’ impairment treatment, moreover, is highlighted by their high incidence. Considering stroke, which is the first cause of acquired disability worldwide [3], the lack of control and weakness of upper limbs represent the most common impairments caused by the disease [4]. Despite the need for adequate therapies to counteract the effects of acquired physical disabilities, we are now witnessing a reduction in hospital-based treatment duration, with the perspective of a further reduction in the future [5,6]. The increasing life expectance and the higher prevalence of motor disability in older people result in a rising need for resources in healthcare systems [7] that is expected to continuously grow. Moreover, the limited availability of professional therapists [8] does not allow healthcare systems to provide the needed care using the current paradigm. Besides the limited resources, the low accessibility of care services also represents a barrier. Many people experience difficulties in accessing the care services due to distance from the clinical centers [9], and this can hinder their adherence to the rehabilitation program and the effectiveness of the treatment. The aforementioned considerations highlight the increasing need to find new solutions to provide effective rehabilitation, lowering at the same time the cost associated with the service provision and enhancing the accessibility of care. Technology-assisted rehabilitation offers the opportunity to overcome the current limitations, reducing the number of therapists needed to administer the therapy. In particular, telerehabilitation represents a valuable solution, moving the rehabilitation settings from clinics to home, guaranteeing higher accessibility and continuity of care, providing an effectiveness equal to the traditional therapies [10].

The implementation of any rehabilitation program has to rely on evidence-based factors improving the recovery of neuromotor functionalities, including the execution of task-oriented exercises [11,12], a high number of repetitions [13], engagement and motivation of the patients [14] and customization of the therapy [15]. Several technological solutions including these factors have been investigated. In particular, systems based on robotics and virtual reality have been implemented.

While robotic systems are suitable for patients with limited residual functionalities and allow the patients to perform a large number of repetitions with limited effort from the therapist, their application, especially in home settings, is severely limited by the high costs and the setup difficulties [16]. On the other hand, solutions implementing virtual environments for providing rehabilitation have also been developed, using different technologies to monitor and evaluate the execution of the exercises. Among them, approaches leveraging consoles for video gaming have been explored. These systems use platforms such as the Nintendo Wii or Xbox Kinect to develop games aimed at providing rehabilitation exercises, but they present excessive difficulties in the use by the patients, and they are not yet adjustable to the patient’s needs [17]. Fully immersive virtual reality systems have also been used to provide rehabilitation therapy, but their use is limited by the required effort in setting up the environment and the high costs of the hardware [18].

To overcome the limitations of the systems currently available, we explored the possibility of using radio frequency identification (RFID) technology to identify the interactions between the patient and objects during the execution of reaching and grasping tasks [19], with the aim of guiding the patient toward rehabilitation exercises and monitor their execution. The translation of RFID to the rehabilitation context required an accurate optimization phase to adapt the characteristics of this technology to the needs of the application, as well as a novel approach to extract descriptive metrics for the available signal properties. In the following sections, we will show the process implemented to adapt a mainstream technology, i.e., RFID, to the requirements of an upper limb rehabilitation system and the related results. This process starts from the hardware implementation of the system, taking into account both hardware performance requirements and the impact on the usability of the system experienced by the patients. We will then move to the optimization and tuning of the characteristic parameters of the communication protocol leading to a robust data acquisition. Finally, we will show the strategy and the algorithms implemented to extract performance metrics of the execution of the exercises from the analysis of the radiofrequency (RF) signals. This includes devoted methods for computing the execution time of the tasks and estimating path length and velocity using RF signals.

## 2. Materials and Methods

### 2.1. System Overall Description

The system presented in this paper relies on the use of passive ultra-high frequency (UHF) RFID technology. These RFID systems are composed of three elements: one or more tags, a reader, and a backend. The tags are small devices comprising a microchip for data storage and communication functionalities provision and an antenna. Passive tags are able to obtain the power needed to communicate from the electromagnetic waves emitted by a reader. The reader is a component that uses RF to read and write data of the tags. It emits RF signals through an antenna to query the tags, collects the responses and sends the data to a backend device. Finally, the backend is in charge of storing and processing the information received by the reader and controlling the reader’s operations.

**Figure 1:**
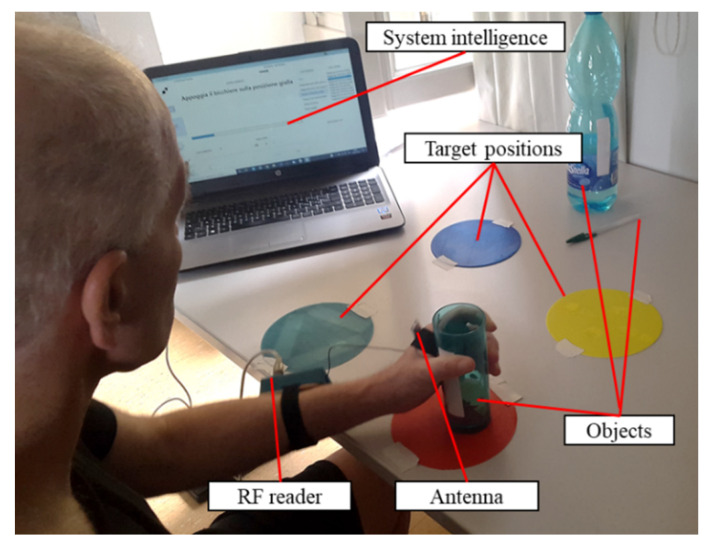
System worn by a patient during its usage. The patient wears the reader on his forearm and the antenna on his hand. The target positions and the objects are equipped with the tags and placed in the workspace. The system intelligence runs on a devoted PC.

We have used this architecture and its components to develop a rehabilitation system for the upper limb, that allows the identification of patient–object interactions and guides the patient toward the execution of physical exercises (Figure 1).

In particular, the patient is asked to wear the reader on his/her arm, with its antenna positioned on the hand. The tags are attached to the objects or on target positions used during the rehabilitation therapy to uniquely identify them. The system intelligence, running on a PC, receives and elaborates the data from the reader and implements a graphical user interface (GUI) to guide the patient through the execution of the exercises. The therapy consists of the execution of functional exercises, reproducing ADLs (e.g., grasping and moving a glass, picking up a pen, taking a bottle). The exercises are composed of simple sequences of reaching, grasping, and moving tasks, involving the tagged objects and target positions, that the patient is instructed to accomplish in order to complete the exercise. The system prompts each task to be executed through the GUI, giving visual and vocal instructions to the patient, and monitors whether the task has been successfully executed. Upon each task completion, the next task to be executed in the sequence is automatically prompted by the GUI. The procedure goes on until the sequence of tasks composing the exercise is completed. To verify the correct execution of each task, the received signal strength indicator (RSSI) of the signal received from the tag, which identifies the object or the space landmark corresponding to the target to be reached, is elaborated, as described in [19]. The elaboration of the RSSI allows for the identification of the contact between the patient’s hand and the target, thus indicating the completion of the task. During the execution of the tasks, the data collected by the reader are continuously recorded not only for the estimation of the task completion event but also for quantifying the execution performances as described in the following Section 2.3.

The exercises to be accomplished by the patient are designed by the therapist. Indeed, the GUI has been developed to allow the design and implementation of custom exercises as sequences of tasks, using personal objects. Through this procedure, the therapist can associate a tag to the objects that best fit the needs of the user, making them recognizable by the system and, therefore, part of the system itself. Moreover, specific sequences can be created, with custom levels of difficulty.

To fully leverage the RFID technology in the implementation of the described rehabilitation system, the components constituting the RFID architecture (i.e., reader and antenna, tag, backend) were carefully chosen. The first chosen component is the reader. An RFID reader operating in UHF is needed, able to use bandwidth compliant with the European Union (EU) regulations. Then the communication parameters, that will be fully described in Section 2.2, have to be fully settable to tune the reading operations according to the needs of our application. Moreover, the signal’s RSSI and phase have to be accessible for all the read tags. Finally, the size and the weight of the device have to be suitable to be mounted on the patient’s forearm, without interfering with the limb’s movement. The M6e-micro (ThingMagic, Woburn, MA, USA) was deemed fully compliant with the aforementioned requirements, allowing transmission in the EU bandwidth, customization of the transmission parameters, and presenting suitable physical properties (i.e., length 69 mm, width 43 mm, height 15 mm, weight 34 g).

The second element to be selected is the reader’s antenna. Since the antenna has to be mounted on the patient’s hand during the execution of the exercises, its shape factor and wearability are key drivers in the choice of the component. For this reason, a flexible dipole antenna (ISM Dipole flexible antenna, Molex, Lisle, IL, USA) with small dimensions (length 87.4 mm, width 12.4 mm, height 0.25 mm, weight 0.745 g) was selected. The antenna has a linear polarization and 1.2 dBi gain, where dBi is the measure of the gain with respect to an isotropic antenna.

Then, the tags were selected. The most suitable were deemed to be the UH101 (Lab-ID, Castel Maggiore, Italy), mounting a UCode 8 chip (NXP, Eindhoven, The Netherlands) and printed on wet inlay to allow an easy application to the objects.

Finally, the backend, including the system intelligence, is represented by software running on a PC, to which the reader is connected via USB. The software implements the GUI that has been previously described, manages all the communications with the reader and processes the acquired data

Since the system implies that the patient wears the reader on his/her arm and the antenna on his/her hand to correctly determine the patient-object interactions, the comfort in the use of the developed hardware needs to be assessed. This aspect is of paramount importance to guarantee the full transferability of the concept to real use in clinical practice, as poor usability results in a low adherence and actual use of the system [20]. To this purpose, the performance of the developed system in terms of usability and acceptability was evaluated. The system was presented to a sample of 10 post-stroke patients. They were asked to wear and use the system to perform three rehabilitation sessions of 30 min each. At the end of the last session, the patients evaluated the usability of the system utilizing the System Usability Scale (SUS), consisting of ten questions on a 5-point Likert scale ranging between 0 (no satisfaction) and 100 (extreme satisfaction) [21].

### 2.2. Communication Protocol Adaptation

**Figure 2:**
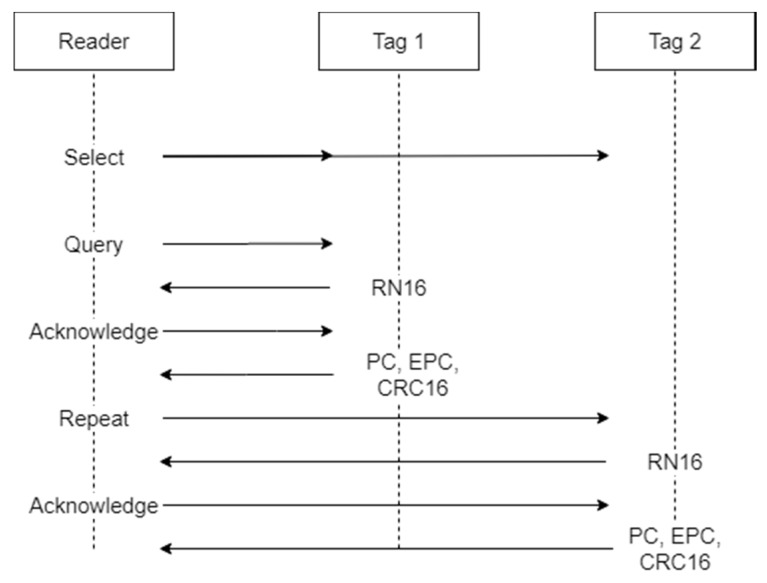
Basic steps of the EPC-Global Class-1 Gen-2 communication protocol. In the figure, an example of the interaction between a reader and two tags is depicted.

The protocol used to regulate the communication between the reader and the tag is the EPC-Global Class-1 Generation-2 [22]. This protocol has been adopted as an international standard by ISO/IEC and determines the procedures that have to be implemented for the communication between reader and tags in the UHF RFID systems. The basic operational steps envisaged by the protocol are shown in Figure 2.

The communication between the reader and the tag is performed during an inventory round. During the inventory, the reader activates all the tags available in its reading range, or part of them, communicates the parameters they have to use to respond, and receives the tags’ responses. Multiple inventories may be performed consecutively to have multiple readings of the tags. The first operation performed by the reader in the inventory round is sending a “Select” command. This command is used to select the population of tags that will be allowed to respond to the reader during the inventory. In the “Select” command, the parameters that each tag has to match to be allowed to respond are sent, and they may be represented by the Electronic Product Code (EPC), or part of it, as well as a piece of custom information in the tag’s memory content. Only the tags that match the selection criteria will be active during the subsequent phases. The next step is a “Query” command. The “Query” command determines the parameters to be used during the inventory round for the tag to reader communications. Such parameters include:

- Backscatter link frequency (BLF): the frequency of the subcarrier wave for data codification in the response of the tag to the reader.
- Type-A reference interval (Tari): the temporal length of a “0” symbol in the tag’s response.
- Data encoding: logic for “0” and “1” symbol codification in the tag response. Two different codifications may be used, the FM0 baseband and the Miller modulation. While FM0 encoding uses a baseband phase inversion between each symbol and an additional phase inversion in the middle of the symbol for “0” encoding, the Miller modulation uses multiple phase inversion cycles to encode the symbols. Depending on the number of cycles used, the codification may be Miller-2, Miller-4 or Miller-8.
- “Q” value: as multiple tags may be allowed to respond to an inventory round, the expected response time is divided into multiple slots, and each tag is allowed to send its response in only one of them, by choosing a random number between 0 and the maximum number of slots, where the maximum number of slots is equal to 2^Q^.

At each iteration of the query, the slot counter of the tags decreases by one unit, and if it is equal to 0, the tag will respond to the query. In this case, the tag generates a 16-bit random number (RN16) and sends it to the reader. If no collision occurs (i.e., just one tag sends the RN16), the reader receives the RN16 and sends an “Acknowledgment” message to the responding tag, containing the same random number. If the tag receives the “Acknowledgment” and the contained number matches the RN16, the tag sends its EPC, a cyclic-redundancy check (CRC) and a protocol-control (PC). The EPC is the unique identifier of the tag and represents the most important information communicated by the tag to the reader. If other operations are required by the reader, such as accessing specific memory bank to read or write data, a handle is also sent by the tag, and an “Access” command is issued by the reader. At the end of this process, a “Repetition” command is issued, and the tags in the following slot will respond. This cycle is repeated until the total number of slots has been consumed and, therefore, the inventory round has finished. Besides the communication parameters, the maximum time during which the reader is allowed to perform the inventories may also be set.

It is clear that the parameters used to control the communication protocol and the reading severely affect the performance of the system. Thus, the first step toward the optimization of the system has been the determination of the best combination of parameters, allowing the maximization of the detection frequency of a single tag while reducing the consumed power. The frequency control process is needed in order to be able to correctly analyze the arm movements during the execution of reaching tasks. On the other hand, the minimization of power consumption is important to reduce the heating of the device and, therefore, to avoid discomfort and burning during the device usage.

To increase the tag detection rate, the first parameter to consider is the “Q” value. Indeed, if the number of slots is too high in relation to the number of tags, the time required by the reader to perform an inventory could be oversized. As in each task to be accomplished during the execution of the exercises only one tag is used to identify the target, the optimal number of slots would be one. On the other hand, the other tags present in the environment that identify the subsequent targets need to be considered to avoid collisions and loss of responses. To optimize the reading, we used the “Select” command to allow only the tag identifying the current target to respond. Since the tag’s EPC is known by the system and it is programmed to be unique among the tags used to perform the exercises, the “Select” command is programmed to activate only the tags with an EPC corresponding to the desired one. Using this strategy, the response of only one tag is assured for each inventory cycle, and the “Q” value can be set to 0 to have only one slot.

In order to choose the best combination of transmission parameters (i.e., Tari, BLF, M value), experimental trials were conducted, placing the tag and the reader’s antenna at a distance of 50 cm one from the other. In order to reproduce the working conditions (i.e., antenna worn on patient’s hand), a hand-shaped container filled with 150 mL of a solution of water and NaCl (9 g/L) was placed in contact with the antenna, between the antenna and the tag. Any possible combination of parameters was applied and, for each combination, 100 inventory rounds were performed using a +30 dBm transmission power. The procedure was repeated using reading times from 4 ms (minimum settable time) up to 10 ms. The percentage of correct readings for each combination of parameters and activation time was computed.

During the trials, no readings were recorded using a BLF equal to 320 and 640 kHz; therefore, only the results obtained using a BLF equal to 250 kHz are reported for each possible Tari value in Table 1, Table 2 and Table 3.

The results show that the encoding typology represents the parameter with the main impact on the reading performance. Indeed, using FM0 encoding, it was not possible to read the tag for all the possible configurations of BLF and Tari. Using Miller encoding improves the capability to read the tag, and, in particular, using Miller-4 and Miller-8 encoding results in a reading rate of 100% for all the activation times imposed and available parameter combinations. The application of Miller encoding forces the use of a BLF equal to 250 kHz, as higher frequencies only support the use of FM0 encoding. As all the available Tari values in combination with Miller-4 and Miller-8 encoding show the same reading rate, the actual reading frequency was investigated during continuous reading cycles. To this aim, the same setup used in the previous experiment was used. A continuous reading was set, with 4 ms of active transmission and 26 ms of inactivity. All the combinations of the Tari values with Miller-4 and Miller-8 encoding were tested. The tag detection frequency was computed and the results are reported in Table 4.

The results highlight that the use of Miller-8 encoding shows lower detection frequencies for all the Tari values, and that higher Tari values, on the other hand, reduce the detection frequency. As expected, shorter Tari and Miller-4 require lower reading times, while increasing the Tari and Miller values required an actual reading time longer than the nominal one. In order to minimize the reading time, the following parameters were selected: Miller-4 encoding, 6.25 µs Tari, 250 kHz BLF.

The subsequent parameter to be set is the duty cycle, intended as the percentage of the active transmission time with respect to the sum of active transmission and inactivity time. The duty cycle determines the acquisition frequency and impacts the power consumption and heating of the reader. In order to choose the best combination of active and inactive time, the desired acquisition frequency was estimated. The wavelength at the used frequency (867.5 MHz) is equal to 0.35 m. Since the phase signal provided by the reader ranges between 0° and 180°, and at least two points need to be sampled for each phase period, a sample needs to be acquired every 0.087 m of distance change. As the peak velocity during reaching tasks in healthy subjects is normally around 0.7 m/s [23], the maximum time distance between two samples was 0.12 s, corresponding to 8.3 Hz. In order to account for higher velocities, and improve the signal quality, a minimum frequency of 30 Hz was chosen. To obtain the desired frequency, the minimum reading time of 4 ms was set in combination with 26 ms of inactive time, corresponding to a duty cycle of 13%. The time parameters set results in a 33 Hz frequency, to account for the tolerance in the hardware time settings, that could prolong the actual reading and inactive times.

### 2.3. Measures Extracted

The signal received by the reader is characterized by two parameters, besides the information on the tag identification: the RSSI and the phase (φ). The RSSI quantifies the power level of the signal received by the reader from a responding tag, and it is defined as [24]:

(1) $$RSSI={\left(\frac{\lambda }{4\pi D}\right)}^{4}\cdot G_{t}^{2}\cdot G_{r}^{2}\cdot {\left|1+\sum H_{i}\right|}^{4}$$

where *λ* is the wavelength, *D* is the transmitter-receiver distance, $G_{t}$ is the gain of the tag antenna, $G_{r}$ is the gain of the reader antenna, $H_{i}$ is the contribution of the *i-*th multipath.

The received signal phase can be defined as [25]:

(2) $$\varphi =mod\left(\frac{-4\pi }{\lambda }D+\varphi_{0},\pi \right)$$

where *mod()* is the modulus function and $\varphi_{0}\;$is the phase offset.

During the execution of the exercises, the RSSI and the phase are recorded alongside the timestamp relative to the received tag response. This information can be used to extract meaningful measures about the performance of each task composing the exercise.

A performance measure is the movement time of the hand. It was mentioned before that the task completion is automatically detected through the RSSI value, but the actual execution time cannot be inferred from the task completion timestamp. Indeed, the patient can start the movement after the task instruction has been delivered, with a delay that is not controllable, highlighting the need for a precise identification of the actual starting and stopping time of the hand movement. To this aim, we used the time variations of the RSSI and phase signals to identify the time points of the movement’s start and stop. Indeed, RSSI and phase are characterized by significant variations during relative movement between the reader’s antenna and the tag. These variations are due to the changes in the relative distance between the two devices, which determines both the RSSI and phase value, as shown in (1) and (2). Nonetheless, smaller signal oscillations are present even if the relative distance does not change, due to noise. The proposed approach, therefore, is aimed at distinguishing the noise signal oscillation from the movement-related variations.

**Figure 3:**
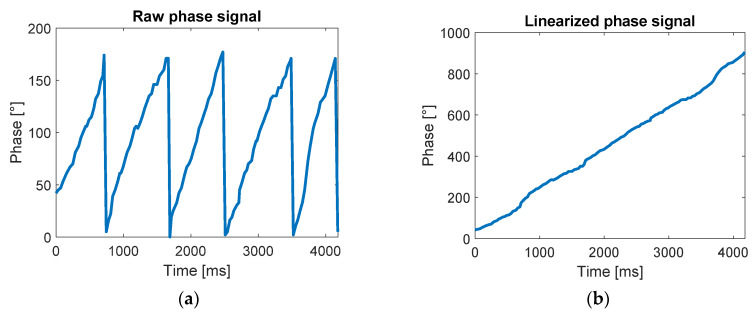
The figure shows the unwrapping of the signal: (**a**) shows the raw phase signal during the movement of the antenna toward the tag and (**b**) shows the same signal after the application of the unwrapping. The signal shown was acquired during the linear movement of the antenna toward the tag. The tag was fixed to a support, while the antenna was moved by hand on a rectilinear path toward the antenna.

As shown in (2), the phase signal is a periodic signal ranging from 0° to 180°. This aspect highly impacts the phase variation calculation as a small oscillation around 0° and 180° may result in large apparent variations (e.g., a decrease of −10° from 5°, that is, 5° − 10° = −5°, would result in a difference of 170°). To solve this problem, a signal unwrapping process was applied, as described in [26], to eliminate the signal periodicity as shown in Figure 3.

The variation range has been then computed for each time point both for the RSSI and phase according to the following equations:

(3) $$Rp_{i}=max\tilde{\varphi }-min\tilde{\varphi }$$

(4) $$Rr_{i}=max\tilde{RSSI}-min\tilde{RSSI}$$

where *Rp_i_* and *Rr_i_* are the range of the phase signal and of the RSSI signal, respectively, on the *i*-th sample,$\;\tilde{\varphi }=\varphi_{1},\ldots ,\;\varphi_{i}$ is the subset of the phase signal samples from the first to the *i*-th, and $\tilde{RSSI}=RSSI_{1},\ldots ,\;RSSI_{i}$ is the subset of the RSSI signal samples from the first to the *i*-th.

The movement start time is identified as the first sample where *Rp* is higher than 5° and *Rr* is higher than 1 dBm. The threshold for the phase and RSSI range was chosen as the maximum range value recorded during static trials (i.e., the antenna and the tag are not moving). The identification of the end of the movement was implemented by checking the signals variations during time intervals of 200 ms. The introduction of a time interval constraint is necessary to take into account the small variations produced by slow movements. Indeed, a variation under the threshold in a short time period may not be due to the absence of movement, but to a short distance covered. Thus, the range of phase and the range of RSSI were computed in 200 ms-long time intervals, and the movement end time was identified as the first point for which the phase range and the RSSI range remained lower than 5° and 1 dBm, respectively, for a time period of at least 200 ms.

**Figure 4:**
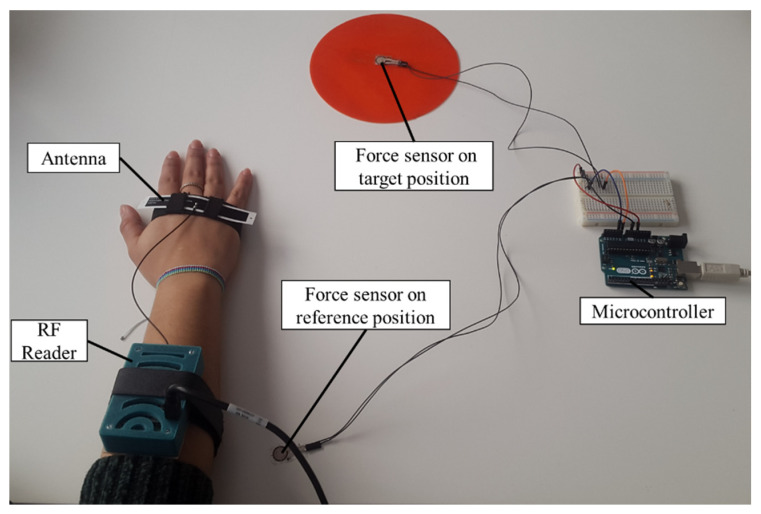
The experimental setup for the comparison of the radiofrequency (RF) signal al the force sensors’ signal. The target and reference positions, the RF system and the microcontroller are depicted.

To validate the approach described to determine the start and stop time of the movements, the results obtained with the analysis of RF signal were compared with force sensors. The validation tests involved a subject wearing the system, seated in front of a table where a reference position was placed in front of the subject, and a target (i.e., a position on the table or an object) was placed 40 cm away from the position. During the test, the subject had to perform four different types of reaching task, resembling the ones proposed during the execution of the rehabilitation exercises: reaching the target position starting from the reference position; reaching the reference position starting from the target position, reaching the target object starting from the reference position; reaching the reference position starting from the target object. The positions and the object were equipped with a tag, to be detectable by the system, and with a force sensor (FlexiForce A301, Tekscan, Boston, MA, USA). In this way, the force applied by the subject’s hand was obtained and, therefore, the contact with the position or the object was detected (Figure 4). The force signal was acquired through a microcontroller (Arduino UNO, Arduino, Torino, Italy) with a sampling frequency of 100 Hz and transmitted via serial communication to a PC. Dedicated software was developed to acquire and synchronize the RF signal and the force sensors signals.

**Figure 5:**
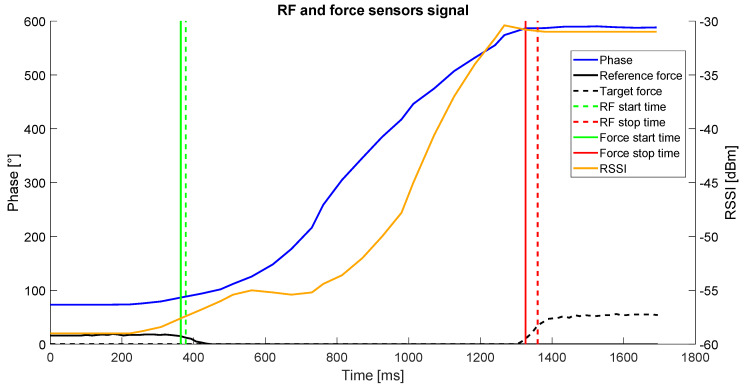
RF and force sensors data acquired during a reaching task. The continuous blue line represents the phase signal; the continuous orange line represents the received signal strength indicator (RSSI) signal; the continuous black line represents the force signal of the reference position; the dashed black line represents the force signal of the target position; the vertical green line and the vertical red line represent the movement starting time and end time respectively identified through the RF signals; the vertical dashed green line and the vertical dashed red line represent the movement starting time and end time respectively identified through the RF signals.

Each task was performed 5 times by each participant. A total number of 4 participants performed the tests. The start and stop time were extracted from the force signal. In this case, the starting time was considered as the first sample for which the force value was below 50% of the maximum value recorded during the trial by the force sensor placed in the starting position. The stopping time was counted as the first sample for which the force value was over 50% of the maximum value recorded during the trial by the force sensor placed in the final position. The start and stop times detected through the force sensors and the RF signal were compared by computing the time difference for each point between the two systems. A sample of the obtained results is shown in Figure 5.

Besides the movement time, the distance covered by the patient’s hand during the execution of a task was computed by the system through the analysis of the received signal. For the computation of the distance covered by a moving antenna with respect to a fixed tag using the phase variations, a proprietary algorithm [26] was applied. Indeed, from (1) and (2), it is evident that both the RSSI and the phase signals are dependent on the distance between the antenna and the tag. Nonetheless, the multipath effect affecting the RSSI [27] and the periodicity of the phase signal, do not allow a fine computation of the antenna-tag distance and, therefore, these relations cannot be used for the estimation of the distance covered. Our approach leverages on the changes in the phase signal. Considering the unwrapped phase signal described above, the variation in the distance between the tag and the antenna occurred between two subsequent samples acquired by the reader during the movement can be estimated by looking at the change in the signal phase through the following equation:

(5) $$d_{i}=\frac{c}{-4\pi f}\left(\varphi_{i+1}-\varphi_{i}\right)$$

where $d_{i}$ is the distance covered in the *i*-th time interval, *c* is the speed of light and *f* is the wave’s frequency.

By summing up all the distance differences during the task execution, the total covered distance can be computed as:

(6) $$L\left(t\right)=\overset{t}{\underset{i=1}{\sum }}d_{i}$$

where *L(t)* is the distance covered at the time *t*. Using the phase differences to evaluate the changes in the tag-antenna distance in short time intervals, has the advantage of preventing the accumulation of the phase error, as the distance variation computed at each time interval only depends on the phase variation occurred between two subsequent samples. Moreover, as the acquisition frequency is around 30 Hz, the time difference between each sample and the next one is ~33 ms. This aspect allows a computation on short time intervals, to properly take into account fast variations not detectable on longer time spans.

Moreover, the velocity value can be directly computed for each time interval from the distance change values according to the following equation:

(7) $$v_{i}=\frac{d_{i}}{\left(t_{i+1}-t_{i}\right)}$$

where $v_{i}$ is the velocity in the *i*-th time interval, $t_{i}$ and $t_{i+1}$ are the time of the *i*-th and the *i+*1*-*th samples respectively.

The described approach was compared against an optoelectronic system, showing reliable results during rectilinear movement of the antenna toward the tag, as detailed in [26].

## 3. Results

**Figure 6:**
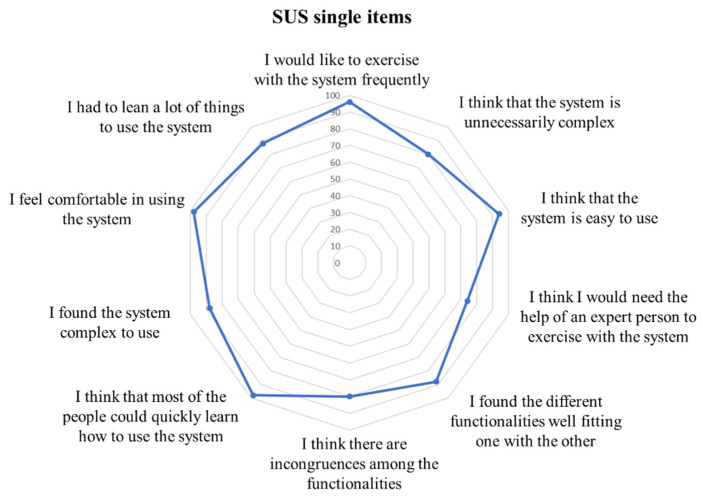
Mean values for each item of the System Usability Scale (SUS) scale in percentage. The highest the value, the more positive the result.

In the previous section, we described the steps implemented to adapt the RFID technology and architecture to the needs of an upper limb rehabilitation system. The components chosen, assembled and developed resulted in a wearable system, mountable on the patient’s forearm and hand, suitable to provide rehabilitation exercises for the upper limb, and to automatically guide the user toward the execution of the therapy. The SUS questionnaire resulted in a mean score of 85.5% ± 13.0%, where 100% represents the maximum score and a score over 68% represents a usability level above average. A detailed overview of the mean scores for each item composing the SUS questionnaire is reported in Figure 6.

The optimization of the communication parameters resulted in an optimal combination of the settings, allowing to obtain reliable readings even in noisy conditions, similar to the ones present in the real-life use, a determined reading frequency equal to 30 Hz, suitable to monitor the execution of reaching tasks, and efficient management of the power consumption, using a 13% duty cycle. The optimal combination was obtained with a BLF of 250 kHz a Tari of 6.25 µs and a Miller-4 encoding. The main driver in the parameters’ selection has been the encoding, where Miller encoding showed better performances, and, in particular, the higher the value the better the performance. This result is in line with the expectations. Indeed, the Miller encoding presents higher robustness against the data loss in a noisy environment by using multiple repetitions in the codification of each symbol, and the higher the Miller value, the higher the number of repetitions. As the testing environment was affected by the presence of water, the use of high Miller values is expected to enhance the performance. Since no difference in successful reading rate was observed between the use of Miller-4 and Miller-8, the second-choice driver is the time frame needed to perform the readings. The Tari value, representing the temporal length of each symbol, highly affects this metric. For this reason, the smallest Tari value was shown to be the most performant. Moreover, as a lower Miller value requires less time to encode the same information because of the lower number of repetitions, Miller-4 resulted in better performance with respect to Miller-8. Finally, the 250 kHz BLF was shown to be the only available choice given the previously described combination of parameters, as higher frequencies do not support Miller encoding.

The results of the estimation of the start and end of the movement are reported in Table 5, where the mean values of the absolute difference between the time point identified by the force sensors and the RF signals are reported for the estimated start and stop time of the movements.

The difference between the times computed using the force sensors and the RF signals shows that the estimation error of start time was lower than 100 ms, while higher errors were obtained in the case of stop time, where mean error values were up to 143 ms.

Concerning the evaluation of the covered distance, it has been previously described and demonstrated by the authors [26] that this estimation can be relatively accurate either for movements of the antenna toward and away from the tag, with a mean estimation error of 8.6 and 28.1 mm, respectively.

## 4. Discussion and Conclusions

The work presented in this paper describes the development and optimization phases of a system that can provide exercises for upper limb rehabilitation leveraging the RFID technology.

The results obtained demonstrate that the process of adaptation of the characteristics of the standard RFID technology allows us to implement a system suitable to be used for upper limb rehabilitation. In particular, the tuning of the reader-tag communication parameters has led to an optimal configuration allowing the transfer of technology for the identification of assets to a robust and fully controllable system able to acquire meaningful data in the context of the movement of the upper limb during the execution of reaching and grasping tasks.

This robust data acquisition system allowed a further step toward the effective use of the technology in the rehabilitation context, providing the basis for the extraction of descriptive measurements for the evaluation of the execution performance. Moreover, the use of the RFID technology allows the immediate association of the movement to a known object, providing the contextualization of the gesture as additional information.

Currently, the RFID technology in the context of rehabilitation has been used to help the therapy provision logistics through indoor localization [28] and for monitoring the activities of patients by detecting the interactions with objects [29,30]. Only one other application that used the RFID technology to drive and monitor rehabilitation exercises has been reported [31]. This application, differently from the system described in this paper, does not allow interactions with objects, but it requires the patient to move along an eight-shaped path, using a cart moved by the patient’s hand. Nonetheless, the use of this technology in the context of rehabilitation may lead to important advantages. First of all, the use of RFID tags allows the execution of the exercises using a potentially unlimited set of objects with different shapes, dimensions, weights and colors. Indeed, all kind of objects can be recognized by the system once they are equipped with a tag, resulting in the possibility to customize the therapy on the patient’s residual functionalities and recovery objectives. Differently from vision-based systems, the introduction of a new object does not require an update of the system with specific training for object recognition. Moreover, the use of real-life objects provides a strong correlation of the rehabilitation exercise presented and the tasks to be performed in daily living. Another important aspect is the portability of the system, that requires only the reader and the antenna to run. The nature of this system allows the execution of the therapy without the need of devoted spaces (a simple table may represent the working space) or fixed devices to be installed (the system can be set up and removed easily before and after the use without any technical expertise). Finally, the easiness of use and the high acceptability reported by the patients highlight the suitability of the system to boost the adherence of the patients to the provided therapy. These considerations, combined with the limited cost characterizing the RFID technology, represent crucial aspects in the perspective of moving the rehabilitation therapy from clinical settings to the home, enhancing the accessibility of patients to the needed rehabilitation therapies, lowering at the same time the burden in terms of costs and effort for the patients and the healthcare systems.

Besides the preliminary results presented in this paper, further steps in the validation process of this system are needed to fully identify the potentialities and limitation of the system. On one hand, the reliability of the measures extracted has to be proof-tested during the system operation in real-life settings during the performance of rehabilitation sessions by the patients. On the other hand, an extensive clinical evaluation has to be conducted on a meaningful sample of patients to further investigate the system usability from the point of view of both patients and clinicians, as well as to evaluate its clinical and cost effectiveness.

## Figures and Tables

**Table 1:** Percentage of recorded readings for each encoding typology and activation time, using a type-A reference interval (Tari) value of 6.25 µs.

On Time [ms]	FM0 [%]	Miller-2 [%]	Miller-4 [%]	Miller-8 [%]
4	0	47	100	100
5	0	50	100	100
6	0	53	100	100
7	0	46	100	100
8	0	44	100	100
9	0	62	100	100
10	0	58	100	100

**Table 2:** Percentage of recorded readings for each encoding typology and activation time, using a Tari value of 12.5 µs.

On Time [ms]	FM0 [%]	Miller-2 [%]	Miller-4 [%]	Miller-8 [%]
4	0	83	99	100
5	0	77	100	100
6	0	75	100	100
7	0	77	99	100
8	0	83	100	100
9	0	77	100	100
10	0	75	99	100

**Table 3:** Percentage of recorded readings for each encoding typology and activation time, using a Tari value of 25 µs.

On Time [ms]	FM0 [%]	Miller-2 [%]	Miller-4 [%]	Miller-8 [%]
4	0	78	100	100
5	0	77	100	100
6	0	70	99	100
7	0	78	99	100
8	0	71	99	100
9	0	79	100	100
10	0	79	100	100

**Table 4:** Sampling frequency obtained for each combination of Tari value and encoding typology.

Tari [µs]	Miller-4 [Hz]	Miller-8 [Hz]
6.25	30.36	27.35
12.5	29.8	27.1
25	28.46	25.71

**Table 5:** Mean ± SD of the error between the start and stop time identified using the force sensors, and the start and stop time computed using RSSI and phase variations.

Task	Start Time Error [ms]	Stop Time Error [ms]
Reach target position from reference position	80.7 ± 64.1	143.3 ± 76.8
Reach reference position from target position	75.5 ± 48.5	94.8 ± 67.8
Reach object from reference position	98.4 ± 72.64	142.6 ± 104.9
Reach reference position from object	79.4 ± 63.7	77.6 ± 65.1

## References

[1] Morris J., Van Wijck F., Joice S., Donaghy M. (2012). Predicting health related quality of life 6 months after stroke: The role of anxiety and upper limb dysfunction. Disabil. Rehabil..

[2] Sveen U., Bautz-Holter E., Sodring K.M., Wyller T.B., Laake K., Sveen E.B.-H.U. (1999). Association between impairments, self-care ability and social activities 1 year after stroke. Disabil. Rehabil..

[3] Mazzoleni S., Duret C., Grosmaire A.G., Battini E. (2017). Combining Upper Limb Robotic Rehabilitation with Other Therapeutic Approaches after Stroke: Current Status, Rationale, and Challenges. BioMed. Res. Int..

[4] Lawrence E.S., Coshall C., Dundas R., Stewart J., Rudd A.G., Howard R., Wolfe C.D.A. (2001). Estimates of the prevalence of acute stroke impairments and disability in a multiethnic population. Stroke.

[5] Maciejasz P., Eschweiler J., Gerlach-Hahn K., Jansen-Troy A., Leonhardt S. (2014). A survey on robotic devices for upper limb rehabilitation. J. Neuroeng. Rehabil..

[6] Richards L., Hanson C., Wellborn M., Sethi A. (2008). Driving Motor Recovery After Stroke. Top. Stroke Rehabil..

[7] Lapchak P., Zhang J.H. (2016). The High Cost of Stroke and Stroke Cytoprotection Research. Transl. Stroke Res..

[8] Gupta N., Castillo-Laborde C., Landry M.D. (2011). Health-related rehabilitation services: Assessing the global supply of and need for human resources. BMC Heal. Serv. Res..

[9] Tchero H., Tabue-Teguo M., Lannuzel A., Rusch E., Abushouk A.I., Dama M., Jacob C., Poder T. (2018). Telerehabilitation for Stroke Survivors: Systematic Review and Meta-Analysis. J. Med. Internet Res..

[10] Rintala A., Päivärinne V., Hakala S., Paltamaa J., Heinonen A., Karvanen J., Sjögren T. (2019). Effectiveness of Technology-Based Distance Physical Rehabilitation Interventions for Improving Physical Functioning in Stroke: A Systematic Review and Meta-analysis of Randomized Controlled Trials. Arch. Phys. Med. Rehabil..

[11] Veerbeek J., Van Wegen E., Van Peppen R., Van Der Wees P.J., Hendriks E., Rietberg M., Kwakkel G. (2014). What Is the Evidence for Physical Therapy Poststroke? A Systematic Review and Meta-Analysis. PLoS ONE.

[12] Barros J., Tani G., Corrêa U.C. (2017). Effects of practice schedule and task specificity on the adaptive process of motor learning. Hum. Mov. Sci..

[13] Pollock A., Farmer S.E., Brady M.C., Langhorne P., Mead G.E., Mehrholz J., Van Wijck F. (2014). Interventions for improving upper limb function after stroke. Cochrane Database Syst. Rev..

[14] Kwakkel G., Wagenaar R.C. (1999). Stroke rehabilitation. Lancet.

[15] Boland L., Légaré F., Perez M.M.B., Menear M., Garvelink M.M., McIsaac D.I., Guérard G.P., Emond J., Brière N., Stacey D. (2017). Impact of home care versus alternative locations of care on elder health outcomes: An overview of systematic reviews. BMC Geriatr..

[16] Kowalczewski J., Prochazka A. (2011). Technology improves upper extremity rehabilitation. Prog. Brain Res..

[17] Mekbib D.B., Han J., Zhang L., Fang S., Jiang H., Zhu J., Roe A.W., Xu D. (2020). Virtual reality therapy for upper limb rehabilitation in patients with stroke: A meta-analysis of randomized clinical trials. Brain Inj..

[18] Tieri G., Morone G., Paolucci S., Iosa M. (2018). Virtual reality in cognitive and motor rehabilitation: Facts, fiction and fallacies. Expert. Rev. Med. Devices.

[19] Baccinelli W., Molteni F., Bulgheroni M. (2018). Smart Objects in Rehabilitation. Proceedings of the Converging Clinical and Engineering Research on Neurorehabilitation II.

[20] Tyagi S., Lim D.S., Ho W.H., Koh Y.Q., Cai V., Koh G.C., Legido-Quigley H. (2018). Acceptance of Tele-Rehabilitation by Stroke Patients: Perceived Barriers and Facilitators. Arch. Phys. Med. Rehabil..

[21] Brooke J. (1996). SUS-A quick and dirty usability scale. Usability Evaluation in Industry.

[22] Global E. (2005). Specification for RFID Air Interface–Radio Frequency Identity Protocols Class 1 Generation 2 UHF RFID, Protocol for communication@ 860-960 MHz.

[23] Wu C., Trombly C.A., Lin K., Tickle-Degnen L. (2000). A kinematic study of contextual effects on reaching performance in persons with and without stroke: Influences of object availability. Arch. Phys. Med. Rehabil..

[24] Marrocco G., Di Giampaolo E., Aliberti R. (2009). Estimation of UHF RFID Reading Regions in Real Environments. IEEE Antennas Propag. Mag..

[25] Martinelli F. (2015). A Robot Localization System Combining RSSI and Phase Shift in UHF-RFID Signals. IEEE Trans. Control. Syst. Technol..

[26] Baccinelli W., Bulgheroni M., Farinelli V., Frigo C.A. (2019). Estimating Hand Kinematics in Reaching Tasks using RFID. A Preliminary Study. Proceedings of the 2019 E-Health and Bioengineering Conference (EHB).

[27] Nikitin P., Rao K. (2008). Antennas and Propagation in UHF RFID Systems. Proceedings of the 2008 IEEE International Conference on RFID.

[28] Wang C.-S., Hung L.-P., Yen N.Y. (2015). Using RFID Positioning Technology to Construct an Automatic Rehabilitation Scheduling Mechanism. J. Med. Syst..

[29] Du Y.G., Lim Y., Tan Y. (2019). A Novel Human Activity Recognition and Prediction in Smart Home Based on Interaction. Sensors.

[30] Barman J., Uswatte G., Sarkar N., Ghaffari T., Sokal B. (2011). Sensor-enabled RFID system for monitoring arm activity in daily life. Proceedings of the 2011 Annual International Conference of the IEEE Engineering in Medicine and Biology Society.

[31] Chen C.-C., Chen Y.-L., Chen S.-C. (2016). Application of RFID technology—Upper extremity rehabilitation training. J. Phys. Ther. Sci..

